# Piezo1 accelerates osteoarthritis progression *via* promotion of HBB-dependent oxidative phosphorylation

**DOI:** 10.1016/j.jbc.2026.113489

**Published:** 2026-08-25

**Authors:** Hongyu Mai, Peichang Yang, Yihang Zhou, Yuanyuan Zhou, Wenjie Hou, Weijia Zhang, Rui Hou, Wanqin Wang, Baoyi Liu, Ningji Gong, Jiali Wang

**Affiliations:** 1 School of Biomedical Engineering, Shenzhen Campus of Sun Yat-sen University, Shenzhen, Guangdong, P. R. China; 2 School of Agriculture and Bioengineering, Foshan University, Foshan, P. R. China; 3 Department of Emergency, The Second Qilu Hospital, Cheeloo College of Medicine, Shandong University, Jinan, Shandong, P. R. China; 4 Nam Yue Natural Medicine Co, Ltd, Macau, P. R. China; 5 Department of Orthopedics, Affiliated Zhongshan Hospital of Dalian University, Dalian, Liaoning, P. R. China; 6 Contributed equally to this work

**Keywords:** Piezo1, osteoarthritis, HBB, energy metabolism, ROS

## Abstract

The mechanosensitive ion channel Piezo1 serves as a key regulator of osteoarthritis (OA) progression, but the underlying mechanism remains largely unknown. In the present study, Piezo1 was found to be upregulated in the articular cartilage of mice with post-traumatic (PT), caused by destabilization of the medial meniscus (DMM) or anterior cruciate ligament transection (ACLT) surgery, or obesity-induced OA, but not in age-related OA. This elevated Piezo1 expression triggered a signaling cascade *via* the KDM6B/KLF1 axis, leading to increased expression of hemoglobin subunit beta (HBB) in chondrocytes. The upregulation of HBB initially enhanced mitochondrial oxidative phosphorylation (OXPHOS), but subsequently impaired mitochondrial function owing to excessive reactive oxygen species (ROS) production, thereby driving chondrocytes an energy metabolism shift from OXPHOS to glycolysis to meet the energy requirements. Notably, intra-articular injection of cartilage-targeting nanoparticles (CAP-PEI) loaded with siRNA against either Piezo1 or HBB effectively restored mitochondrial OXPHOS capacity and attenuated OA progression in mice. Together, these results demonstrate that Piezo1 exacerbates PT and obesity-induced OA by upregulating HBB through the KDM6B/KLF1 pathway, highlighting the therapeutic potential of targeting this metabolic axis.

Osteoarthritis (OA) represents a debilitating degenerative joint disease and a leading cause of global disability, affecting over 500 million people (1). Of note, OA is increasingly prevalent among younger individuals, especially suffering from trauma in knee joints, including anterior cruciate ligament (ACL) injury and meniscus tears (2). In addition, obesity also significantly increases the risk of developing OA, and two out of three individuals with obesity are likely to develop symptomatic knee OA (3). Either trauma or obesity causes aberrant increase of mechanical stress in articular cartilage (4), thereby leading to overactivation of mechanosensitive Piezo1 channel in chondrocytes (5). Emerging evidence demonstrates that Piezo1 is highly associated with OA progression, which may be attributed to excessive calcium (Ca^2+^) influx (6, 7). This in turn shifts the energy metabolism of chondrocytes to oxidative phosphorylation (OXPHOS) at the early stage of OA (8), ultimately resulting in promoted ROS accumulation in mitochondria and accelerated process of cellular senescence (9). Given that normal articular cartilage is hypoxic, as a consequence of the absence of blood vessels in cartilage, oxygen supply to individual cells within tissues primarily relies on diffusion from surrounding synovial fluid. However, it is not adequate for satisfying oxygen needs in OXPHOS (10). Consequently, healthy chondrocytes utilize glycolysis to generate approximately 75% of total cellular ATP (11). Herein, it may raise a question that what is the oxygen supplier to facilitate OXPHOS in Piezo1-mediated OA chondrocytes?

Most recently, chondrocytes within cartilage have been found to produce massive amounts of hemoglobin (12), traditionally known for oxygen transport in erythrocytes. The intracellular hemoglobin acts as a local oxygen storage to sustain chondrocyte survival over regional hypoxia in cartilage, while deletion of hemoglobin in chondrocytes leads to enhanced glycolysis and extensive cell death (12). Based on this fact, we speculate that hemoglobin may serve as an oxygen reservoir that aberrantly facilitates OXPHOS, disrupting the glycolytic homeostasis of chondrocytes in avascular cartilage. Nevertheless, Piezo1 activation is reported to inhibit H3K27me3 expression (13), a repressive epigenetic mark that silences transcription and functions conversely to H3K4me3 (14). Since the expression of Krüppel-like factor 1 (KLF1), which plays a pivotal role in hemoglobin production as a transcription factor (12), is positively regulated by H3K4me3 level, it indicates that Piezo1 activation in articular cartilage caused by trauma or obesity may favor hemoglobin increase in chondrocytes, thereby facilitating oxygen supply in OXPHOS. This proposed mechanism, however, appears to contrast with the well-documented metabolic shift toward glycolysis observed in OA chondrocytes to meet their energy demands (7, 15, 16).

In the present study, we raised a hypothesis that aberrant expression of Piezo1 in articular cartilage, caused by abnormal mechanical stress, may initially drive chondrocytes a metabolic shift toward increased OXPHOS owing to increased hemoglobin for enhanced oxygen supply capability, which may thereby lead to ROS-induced mitochondrial dysfunction and ultimately make OA chondrocytes primarily rely on glycolysis for ATP production at the advanced OA stage. To verify the abovementioned hypothesis, we first determined Piezo1 expression levels in articular cartilage over time in both post-traumatic (PT) and obesity-related mouse OA models. Then, we profiled the temporal expression patterns of genes and proteins associated with OXPHOS and glycolysis across diverse stages of OA and investigated if they were mediated by Piezo1 activation. Subsequently, we explored the effects of Piezo1 activation on hemoglobin expression and delineated the underlying mechanism behind the modulation pathway. Following this, we investigated the effects of hemoglobin on energy metabolism and OA phenotype in Piezo1-overexpressing chondrocytes. Finally, we evaluated whether knockdown of Piezo1 or hemoglobin in chondrocytes could mitigate OA progression by modulating energy metabolism.

## Results

### Piezo1 expression exhibits temporal changes in articular cartilage across distinct OA stages in mice suffering from trauma or obesity

In order to investigate if abnormal mechanical stress induces Piezo1 expression in articular cartilage during OA progression, trauma, *i.e.* anterior cruciate ligament transection (ACLT) and destabilization of the medial meniscus (DMM), obesity with wheel-running exercise, and age-related OA mice were used in the present work. As shown in Figures 1, *A*, *B* and S1*A*, three OA models, induced by excessive mechanical stress, were successfully constructed in terms of OARSI scores and Col II expression in articular cartilage. As inflammatory cytokines are highly involved in the pathogenesis of OA by promoting catabolic and destructive processes (17), mRNA expression levels of *Il-1β* and *Mmp13* in articular cartilage were determined across distinct OA stages (Fig. 1*D* and S1*D*). Consistently, the expression levels of these inflammatory cytokines increased over time and exhibited significantly higher in the 3 mouse OA models relative to the healthy mice. Since senescent cells are found to be significantly elevated in OA lesions of patients and mice compared with normal individuals (18), the percentage of chondrocytes expressing P16^ink4a^, a biomarker of senescent chondrocytes, was quantified in articular cartilage in the 3 mouse OA models. The percentage of senescent chondrocytes displayed an abrupt increase at the advanced OA stage (Figs. 1*C* and S1, *B*, *C*), indicating chondrocyte dysfunction and exacerbation of OA pathogenesis.

**Figure 1:**
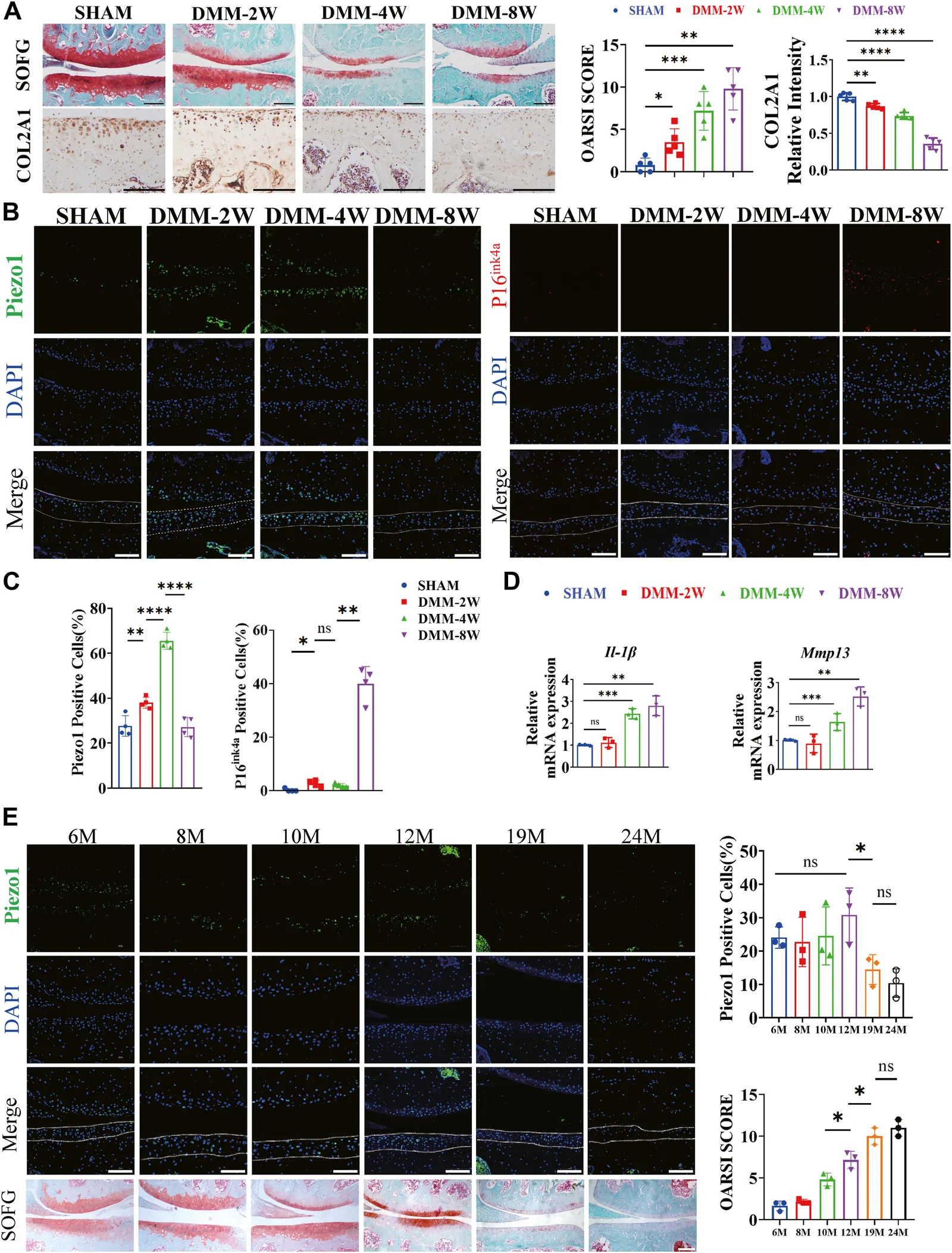
**Piezo1 Dynamics in Traumatic and Aged OA.***A*, Representative Safranin O-Fast Green staining and immunohistochemistry images showing COL2A1 expression at the knee articular cartilage in DMM mouse models at 2, 4, and 8 weeks post-surgery. The sham-operated mice (SHAM) were set as the control. Quantification was performed by analysis of OARSI scores and COL2A1 expression levels. ∗*p* < 0.05, ∗∗*p* < 0.01, ∗∗∗*p* < 0.001 and ∗∗∗∗*p* < 0.0001, n = 5. Scale bar: 100 μm. *B*, representative immunofluorescence images showing Piezo1 and P16^ink4a^ expression at the knee articular cartilage in DMM mouse models at 2, 4, and 8 weeks post-surgery. The SHAM group was set as the control. The outline of tibial cartilage is indicated by a *whitedashed dotted line*. Scale bar: 100 μm. *C*, quantitative analysis of Piezo1 expression and P16^ink4a^ positive cells at the articular cartilage. ns, not significant, ∗*p* < 0.05, ∗∗*p* < 0.01, ∗∗∗*p* < 0.001 and ∗∗∗∗*p* < 0.0001, n = 4. *D*, mRNA levels of *Il-1β* and *Mmp13* of the knee articular cartilage in DMM mouse models at 2, 4, and 8 weeks post-surgery. ns, not significant, ∗*p* < 0.05, ∗∗*p* < 0.01, ∗∗∗*p* < 0.001 and ∗∗∗∗*p* < 0.0001, n = 4. *E*, representative immunofluorescence images showing Piezo1 and representative Safranin O-Fast *Green* staining at the knee articular cartilage in mice at the age of 6, 8, 10, 12, 19, and 24 months. Quantification was performed by analysis of P16^ink4a^ positive cells and OARSI scores. ns, not significant, ∗*p* < 0.05, ∗∗*p* < 0.01 and ∗∗∗∗*p* < 0.0001, n = 4. Scale bar: 100 μm. DMM: Destabilization of the medial meniscus.

Either trauma or obesity may cause abnormal distribution of mechanical stress in articular cartilage (19), which may thereby induce OA progression through modulation of the mechanosensitive Piezo1 ion channel (20). Consistently, overload in articular cartilage of the 3 mouse OA models caused significant increase in Piezo1 expression level within the initial induction period (Figs. 1, *B*, *C* and S1, *B*, *C*). In the early OA phase, articular cartilage is known to be devoid of blood vessels and sensory nerve fibers (21), making knee joint to sense mechanical load without nociceptive input. During mild to severe OA stages, sensory nerves change their distribution and begin to grow within blood vessels intrinsic to the subchondral bone (21), thereby generating pain signals. Reducing mechanical stress stimulating is one commonly behavior to minimize nociceptive pain, so significant decrease in Piezo1 expression, in response to lower mechanical stress, was observed in articular cartilage of the 3 mouse OA models at the late OA stage (Figs. 1, *B*, *C* and S1, *B*, *C*). In contrast, there was no significant increase of Piezo1 expression in articular cartilage of the age-related mouse OA model within 12 months (Fig. 1*E*), indicating that mechanical stress distribution may not be the cause inducing OA onset in spontaneous age-related model. Aging-associated changes that affect articular tissues promote the development of OA. For mice at 19 months, which is equivalent to humans over 65 years old (22), Piezo1 expression in articular cartilage exhibited a remarkable decrease (Fig. 1*E*), which may be ascribed to an aberrant increase of sensory innervation in subchondral bone and thereby causes pain through mechanical sensitization (23). To investigate whether the absence of Piezo1 upregulation in age-related OA was caused by less severe OA pathology, we quantified OA severity using OARSI scores and analyzed the association between Piezo1-positive chondrocyte abundance and OA severity across different OA models (Fig. S2). OARSI evaluation confirmed progressive cartilage degeneration in all OA models, including traumatic/mechanical instability-induced OA models (DMM and ACLT), obesity-associated mechanical overload OA (HFR), and age-related OA. These results demonstrate that aged mice developed evident OA pathology, excluding insufficient disease severity as the reason for absent Piezo1 induction. We further examined the correlation between Piezo1-positive chondrocyte abundance and OARSI scores. In mechanically induced OA models, including DMM (2 and 4 weeks), ACLT (1 and 3 weeks), and HFR (2 and 4 weeks), Piezo1-positive chondrocyte abundance showed a significant positive correlation with cartilage degeneration severity (DMM: Spearman’s ρ = 0.859, *p* = 0.00034; ACLT: ρ = 0.784, *p* = 0.0025; HFR: ρ = 0.917, *p* = 2.67 × 10^−5^). In contrast, aging-associated OA mice (6–12 months) exhibited no correlation between Piezo1 expression and OARSI scores (ρ = 0.0997, *p* = 0.758), despite progressive cartilage degeneration. These findings indicate that Piezo1 upregulation represents an early-to-middle stage response to mechanical instability and excessive loading, rather than a general indicator of OA severity.

### Piezo1 works synergistically with inflammatory cytokines to modulate energy metabolism and phenotype of OA chondrocytes

Since Piezo1-mediated Ca influx may upregulate mitochondrial OXPHOS (24), we quantified expression levels of ATP5A1, lactate dehydrogenase enzyme A (LDHA), and hypoxia-inducible factor 1-alpha (HIF-1α) of articular cartilage in mice following DMM surgery. As evidenced by Western blot analysis and immunofluorescence detection in articular cartilage, the protein level of ATP5A1, which plays a pivotal role in mitochondrial OXPHOS for ATP synthesis, showed significant increase at 2 weeks post-surgery while exhibited a profound decrease at 8 weeks after surgery (Figs. 2, *A*–*C* and S3). Concomitantly, the protein levels of HIF-1α and LDHA, serving as critical regulators orchestrating glycolysis (25), showed significant decrease at 2 weeks post-surgery whereas displayed a significant increase at 8 weeks after surgery. These data demonstrated that OXPHOS in chondrocytes was enhanced at the early OA stage whilst glycolysis in chondrocytes was promoted at the later OA stage.

**Figure 2:**
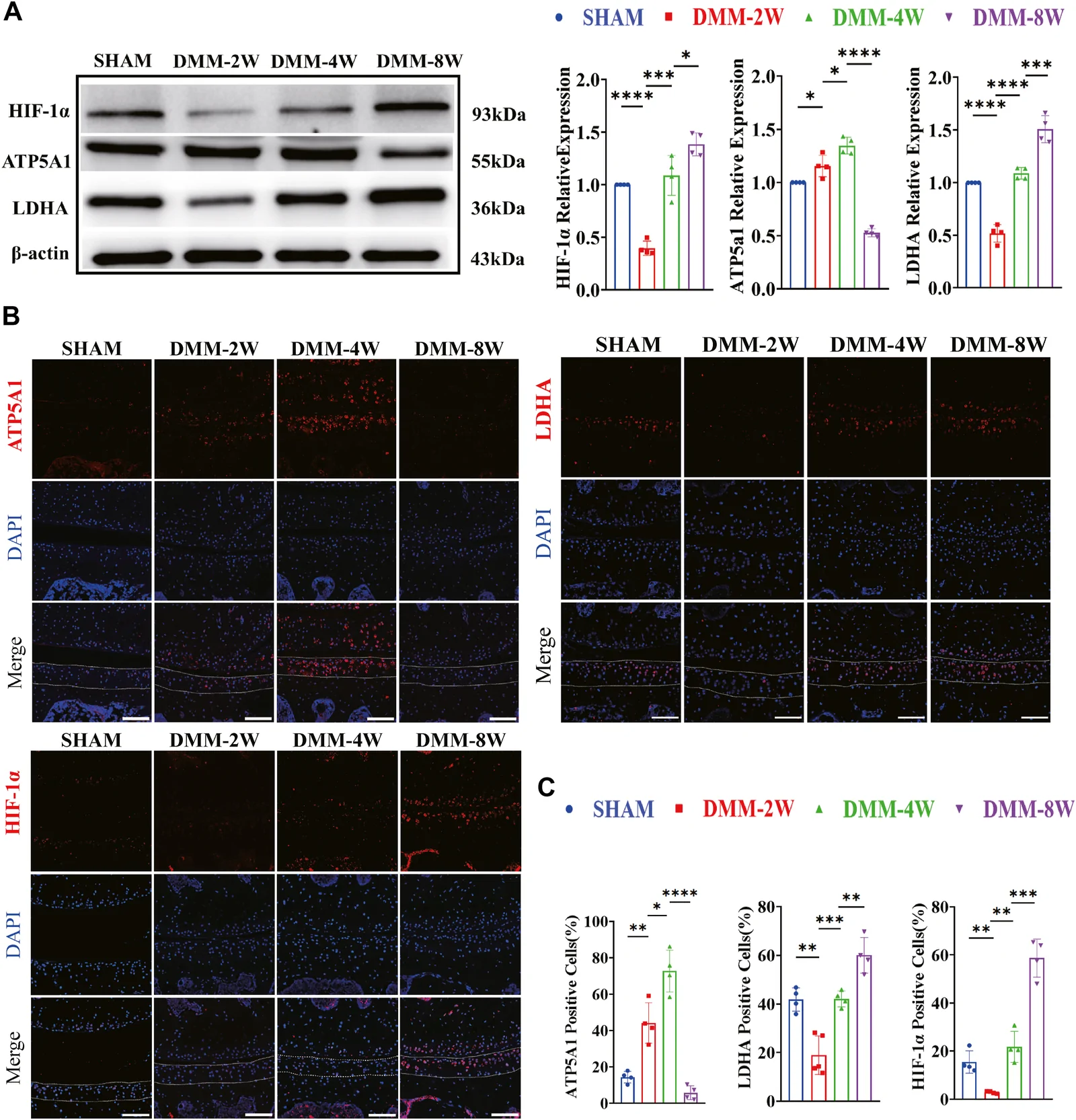
**OXPHOS-to-Glycolysis Shift in OA Cartilage.***A*, Representative Western blot bands and quantitative analysis of ATP5A1, LDHA and HIF-1α at the knee articular cartilage in DMM mouse models at 2, 4, and 8 weeks post-surgery. ∗*p* < 0.05 and ∗∗∗*p* < 0.001, n = 4. *B*, representative immunofluorescence images showing ATP5A1, LDHA and HIF-1α expression at the knee articular cartilage in DMM mouse model at 2, 4, and 8 weeks post-surgery. The outline of tibial cartilage is indicated by a *white dashed dotted line*. Scale bar: 100 μm. *C*, quantitative analysis of ATP5A1, LDHA and HIF-1α positive cells at the articular cartilage. ns, not significant, ∗*p* < 0.05, ∗∗*p* < 0.01, ∗∗∗*p* < 0.001 and ∗∗∗∗*p* < 0.0001, n = 4. Scale bar: 100 μm.

In order to validate the role of Piezo1 in coordinating energy metabolism and OA phenotype, we overexpressed Piezo1 in primary mouse chondrocytes by transfection with a Piezo1-IRES-eGFP plasmid, using an empty PcDNA3.1-IRES-eGFP plasmid as a control (Fig. S4, *A* and *B*). After transfection, the chondrocytes were then treated with the Piezo1 agonist Yoda1 and/or the inflammatory cytokine IL-1β. In terms of OA-related markers, the Piezo1-overexpressing chondrocytes exhibited significantly higher protein levels of P16^ink4a^ and MMP13, but lower levels of Col II, following treatment with IL-1β, Yoda1, or their combination, relative to their respective controls (Fig. 3, *A* and *B*). Notably, in Piezo1-overexpressing chondrocytes, the combined treatment with Yoda1 and IL-1β synergistically enhanced the levels of P16^ink4a^ and MMP13 and further reduced Col II expression relative to either treatment alone. Consistently, SA-β-gal staining analysis validated that Piezo1-overexpressing chondrocytes exhibited significantly higher percentage of SA-β-gal-positive cells, after treatment with IL-1β, Yoda1, or their combination, relative to their respective controls (Fig. 3, *C* and *D*). In addition, in Piezo1-overexpressing chondrocytes, the combined treatment with Yoda1 and IL-1β synergistically increased the percentage of SA-β-gal-positive cells relative to either treatment alone. Collectively, Piezo1 acts synergistically with IL-1β to exacerbate OA phenotype in chondrocytes.

**Figure 3:**
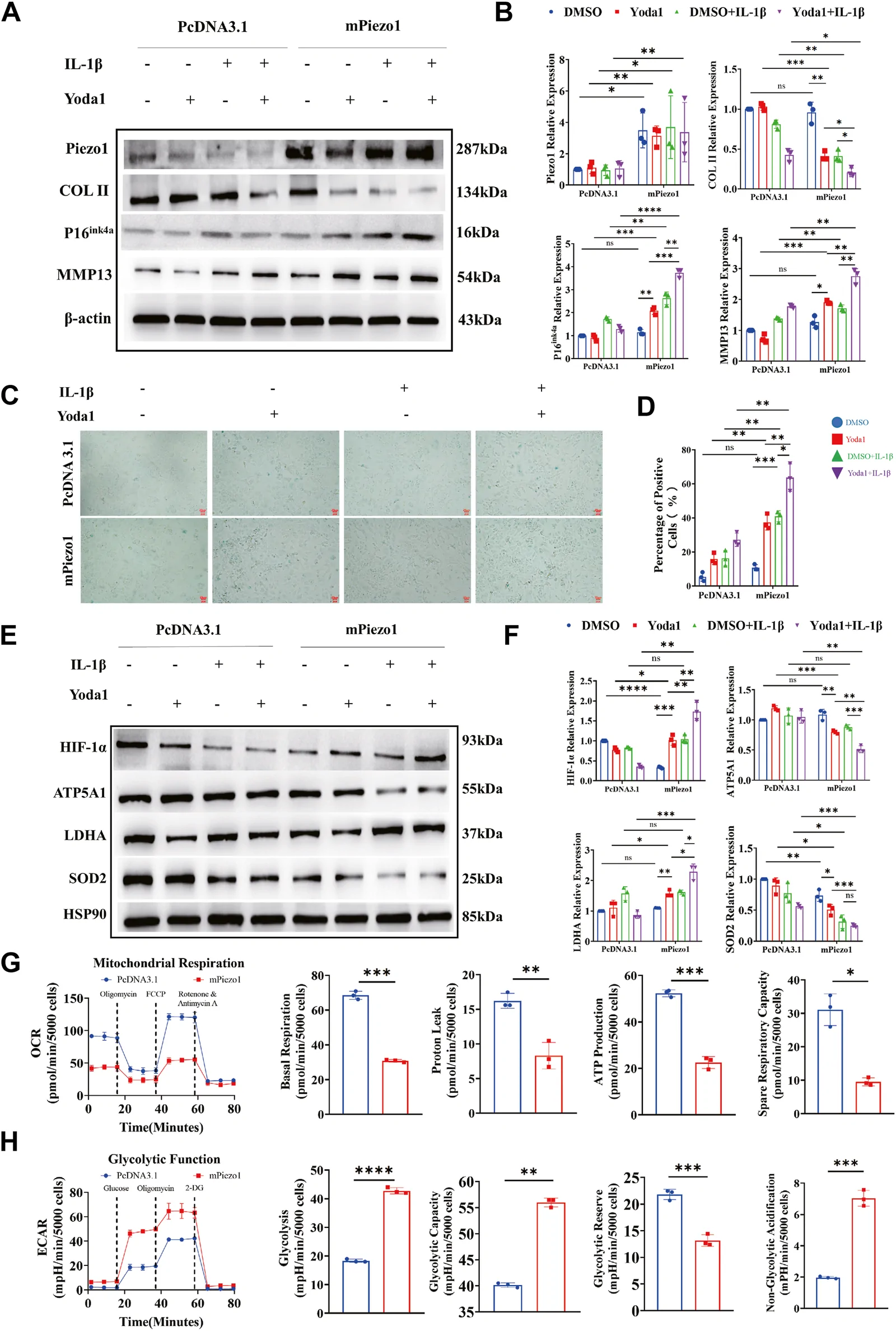
**Piezo1/IL-1β Synergy Drives OA and Metabolic Reprogramming.***A*, Representative Western blot bands of Piezo1, COL II, P16^ink4a^ and MMP13 in the primary chondrocytes transfected with either empty vector (PcDNA3.1-IRES-eGFP) or mPiezo1-overexpression vector after treatment with or without IL-1β and/or Yoda1 for 24 h. *B*, quantitative analysis of Piezo1, COL II, P16^ink4a^ and MMP13 in the primary chondrocytes transfected with either empty vector (PcDNA3.1-IRES-eGFP) or mPiezo1-overexpression vector after treatment with or without IL-1β and/or Yoda1 for 24 h ns, not significant, ∗*p* < 0.05 and ∗∗*p* < 0.01, n = 3. *C*, representative β-Galactosidase staining of the primary chondrocytes transfected with either empty vector (PcDNA3.1-IRES-eGFP) or mPiezo1-overexpression vector after treatment with or without IL-1β and/or Yoda1 for 24 h. *D*, quantitative analysis of β-Galactosidase-positive cell percentage of the primary chondrocytes transfected with either empty vector (PcDNA3.1-IRES-eGFP) or mPiezo1-overexpression vector after treatment with or without IL-1β and/or Yoda1 for 24 h ns, not significant, ∗∗*p* < 0.01 and ∗∗∗*p* < 0.001, n = 3. Scale bar: 100 μm. *E*, representative Western blot bands of HIF-1α, ATP5A1, LDHA and SOD2 in the primary chondrocytes transfected with either empty vector (PcDNA3.1-IRES-eGFP) or mPiezo1-overexpression vector after treatment with or without IL-1β and/or Yoda1 for 24 h. *F*, quantitative analysis of HIF-1α, ATP5A1, LDHA and SOD2 in the primary chondrocytes transfected with either empty vector (PcDNA3.1-IRES-eGFP) or mPiezo1-overexpression vector after treatment with or without IL-1β and/or Yoda1 for 24 h ns, not significant, ∗*p* < 0.05 and ∗∗*p* < 0.01, n = 3. *G*, representative oxygen consumption rate (OCR) profiles and quantitative analysis of mitochondrial respiration parameters, including basal respiration, ATP production, maximal respiration, and spare respiratory capacity in the primary chondrocytes transfected with either empty vector (PcDNA3.1-IRES-eGFP) or mPiezo1-overexpression vector after treatment with IL-1β and Yoda1 for 24 h ns, not significant, ∗*p* < 0.05, ∗∗*p* < 0.01 and ∗∗∗*p* < 0.001, n = 3. *H*, Representative extracellular acidification rate (ECAR) profiles and quantitative analysis of glycolytic parameters, including glycolysis, glycolytic capacity, glycolytic reserve, and non-glycolytic acidification in the primary chondrocytes transfected with either empty vector (PcDNA3.1-IRES-eGFP) or mPiezo1-overexpression vector after treatment with IL-1β and Yoda1 for 24 h ∗∗*p* < 0.01, ∗∗∗*p* < 0.001 and ∗∗∗∗*p* < 0.0001, n = 3.

We next investigated the effects of Piezo1 on energy metabolism of OA chondrocytes. As shown in Figure 3, *E* and *F*, the Piezo1-overexpressing chondrocytes displayed significantly reduced protein levels of ATP5A1 and SOD2, following treatment with Yoda1 or combination of Yoda1 and IL-1β, relative to their respective controls. In addition, the combined treatment with Yoda1 and IL-1β led to a synergistic suppression of ATP5A1 and SOD2 expression relative to either treatment alone. Concomitantly, the Piezo1-overexpressing chondrocytes exhibited significantly higher expression of HIF-1α and LDHA, after treatment with Yoda1 or combination of Yoda1 and IL-1β, relative to their respective controls. To investigate the effect of Piezo1 activation on chondrocyte energy metabolism, we performed Seahorse extracellular flux analysis to assess mitochondrial respiration and glycolytic activity (Fig. 3, *G* and *H*). Compared with the chondrocytes treated by the empty vector, the Piezo1-overexpressing chondrocytes exhibited a significant reduction in mitochondrial oxidative phosphorylation capacity after treatment with Yoda1 and IL-1β for 24 h, as indicated by decreased oxygen consumption rate (OCR). In contrast, extracellular acidification rate (ECAR) analysis revealed that Piezo1 activation enhanced glycolytic activity. These metabolic alterations were accompanied by increased expression of glycolysis-associated molecules, including HIF-1α and LDHA. Together, these findings indicate that Piezo1 activation induces a metabolic shift from mitochondrial oxidative phosphorylation toward glycolytic metabolism in OA chondrocytes.

Collectively, these findings demonstrate that Piezo1 cooperates with IL-1β to induce mitochondrial dysfunction and promote a metabolic shift from oxidative phosphorylation toward glycolysis in OA chondrocytes, thereby facilitating cellular adaptation to increased metabolic demands during OA progression.

### Piezo1 promotes hemoglobin production in OA chondrocytes through activation of KDM6B/KLF1 axis

In normal condition, healthy chondrocytes primarily rely on glycolysis rather than mitochondrial OXPHOS to produce energy (26). Emerging evidence demonstrated that hemoglobin, serving as a local oxygen storage, is essential for the survival of chondrocytes under a hypoxic environment, indicating the crucial role of oxygen supply in tolerance of chondrocytes to hypoxia (12). However, oxygen may exert a double-edged sword effect on chondrocytes. Since mitochondrial dysfunction, mainly caused by excessive production of harmful reactive oxygen species (ROS) (27), is highly associated with OA pathogenesis (28), the amount of hemoglobin may be also a risk factor contributing to OA onset and development. In order to test this hypothesis, the expression levels of hemoglobin-α subunit (HBA) and hemoglobin-β subunit (HBB) were determined in the articular cartilage of mice over time after DMM or ACLT surgery. As shown in Figure 4, *A* and *B*, the percentage of HBB positive chondrocytes significantly increased after 3 and 4 weeks post-surgery in the DMM and ACLT models, respectively, while there were no significant changes of HBA positive chondrocytes. Consistent with the immunofluorescence findings in articular cartilage, HBB protein expression was significantly increased at 2 weeks post-DMM surgery, whereas HBA expression showed no significant change (Fig. 4*C*), indicating that OA pathogenesis is accompanied with upregulation of HBB in the articular cartilage. Interestingly, the expression levels of HBB did not show significant changes at the articular cartilage of mice ranging from 6 to 24 months (Fig. S5). Since Piezo1 plays a pivotal role in trauma- or obesity-induced OA progression, we next examined if Piezo1 influences HBB expression in this process. To further investigate whether Piezo1 regulates HBB expression, Piezo1 was silenced using siRNA in chondrocytes. Western blot analysis confirmed the efficient knockdown of Piezo1 protein expression following siRNA transfection, which was accompanied by a significant reduction in HBB protein levels (Fig. S5*A*). Consistently, immunofluorescence staining of articular cartilage sections from the DMM mouse model demonstrated that Piezo1 knockdown significantly decreased the percentage of HBB-positive chondrocytes at both 4 and 8 weeks post-surgery (Fig. 4, *D* and *E*). These findings indicate that Piezo1 positively regulates HBB expression during OA progression.

**Figure 4:**
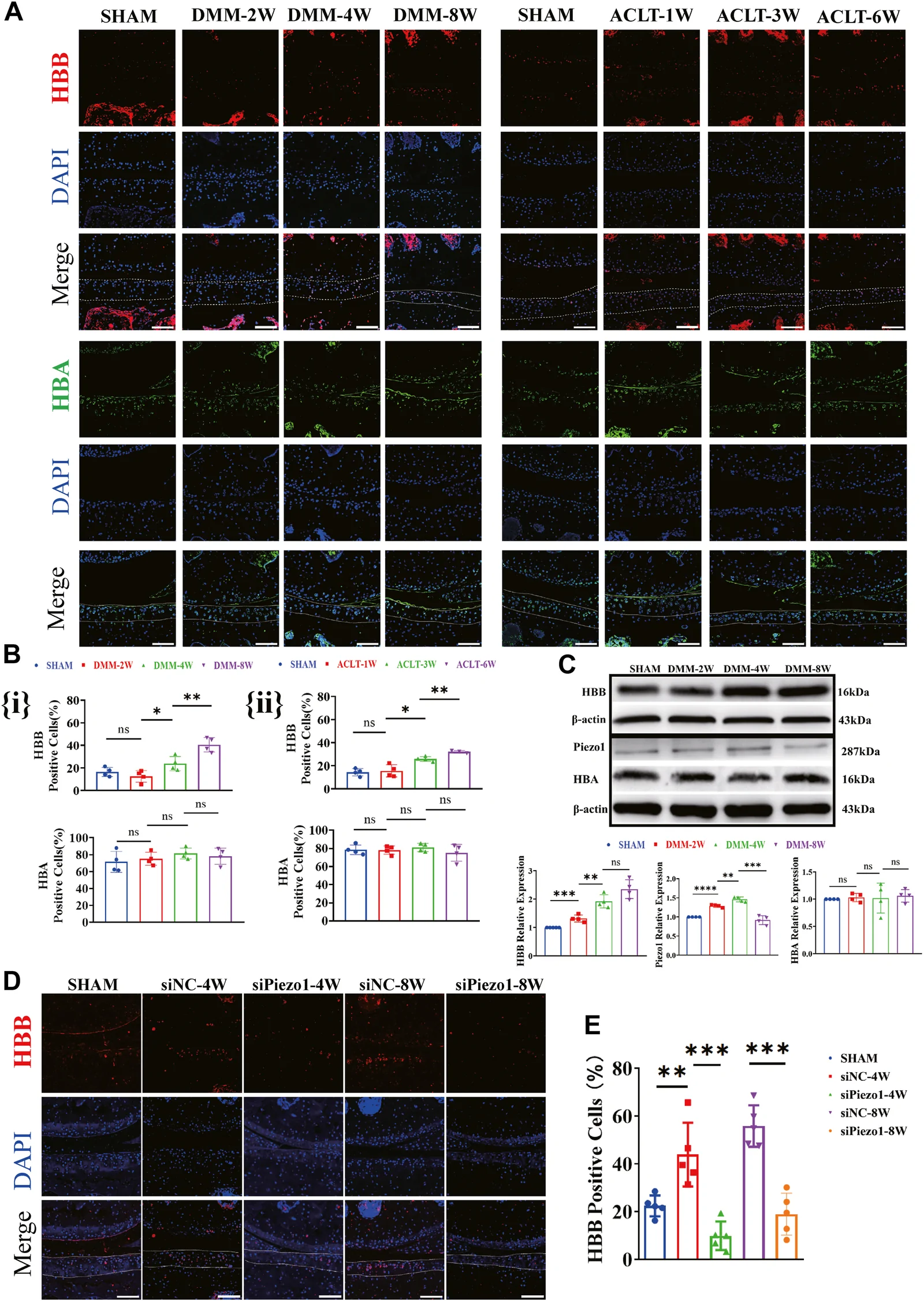
**Piezo1-Dependent HBB Upregulation in OA.***A*, Representative immunofluorescence images showing HBB and HBA expression at the knee articular cartilage in DMM mouse models at 2, 4, and 8 weeks post-surgery and in ACLT mouse models at 1, 3, and 6 weeks post-surgery. The Sham-operated mice were set as the Control. The outline of tibial cartilage is indicated by a *white dashed dotted line*. Scale bar: 100 μm. *B*, quantitative analysis of HBB and HBA fluorescence positive cells at the articular cartilage in DMM mouse model {i} and ACLT mouse model {ii}. ns, not significant, ∗*p* < 0.05, ∗∗*p* < 0.01, ∗∗∗*p* < 0.001 and ∗∗∗∗*p* < 0.0001, n = 4. Scale bar: 100 μm. *C*, representative Western blot bands and quantitative analysis of HBB at the knee articular cartilage from DMM-operated mice at 2, 4, and 8 weeks post-surgery. ns, not significant, ∗*p* < 0.05 and ∗∗∗*p* < 0.001, n = 4. *D*, representative immunofluorescence images showing HBB, Piezo1 and HBA expression at the knee articular cartilage post-surgery in mice receiving CAP-PEI loaded with siNC and siPiezo1. The outline of tibial cartilage is indicated by a *white dashed dotted line*. Scale bar: 100 μm. *E*, quantitative analysis of HBB fluorescence positive cells at the articular cartilage. ns, not significant and ∗∗∗*p* < 0.001, n = 4. Scale bar: 100 μm.

We next sought to elucidate the pathway through which Piezo1 mediates HBB expression in OA chondrocytes. Since Krüppel-like factor 1 (KLF1) plays an important role in the production of hemoglobin (29, 30), the effects of Piezo1 on KLF1 expression in OA chondrocytes were investigated. As shown in Figure 5*A*, the Piezo1-overexpressing chondrocytes showed significant increase of protein levels of KLF1 and HBB relative to their respective controls with or without Yoda1 or IL-1β treatment. Notably, the combined treatment of Yoda1 and IL-1β resulted in a synergistic enhancement of KLF1 and HBB expression levels compared to each individual treatment. Given that lysine demethylase 6B (KDM6B) acts as a crucial enzyme specific modulation of methyl groups from H3K27me3 and H3K4me3 (31), which are highly associated with hemoglobin production (32, 33), their protein levels were further quantified in the chondrocytes with or without overexpression of Piezo1. Consistently, in Piezo1-overexpressing chondrocytes, treatment with Yoda1, IL-1β, or their combination resulted in significantly elevated protein levels of KDM6B and H3K4me3, along with a reduction in H3K27me3, compared to respective controls. Importantly, the combined treatment of Yoda1 and IL-1β led to a synergistic increase of H3K4me3 while decrease of H3K27me3 compared to each individual treatment, which may thereby contribute to improved HBB production (12). In addition, knockdown of Piezo1 reduced the protein expression levels of KDM6B and KLF1 compared with the control group (Fig. 5*B*), indicating that Piezo1 activity is critical for modulation of KDM6B/KLF1 signaling. Given that KDM6B is a histone demethylase responsible for removing repressive H3K27me3 marks, we next investigated whether KDM6B facilitates KLF1 transcriptional activation through epigenetic modification. In a ChIP assay, agarose gel electrophoresis was conducted to confirm the enrichment of KDM6B and H3K27me3 at the Klf1 promoter (Fig. S4*F*). In addition, qPCR analysis revealed a significant increase in KDM6B enrichment while a significant deduction in H3K27me3 enrichment at the Klf1 promoter in OA chondrocytes (Fig. 5*C*). These findings indicate that Piezo1 promotes KLF1 transcription by facilitating KDM6B-mediated demethylation of the KLF1 promoter. To further delineate the role of KDM6B in Piezo1-mediated HBB production, KDM6B was efficiently knocked down in primary chondrocytes, as confirmed by reduced KDM6B expression (Fig. S4*D*). Following combined treatment with Yoda1 and IL-1β, the protein levels of H3K27me3, H3K4me3, KLF1, and HBB were subsequently quantified in chondrocytes with or without KDM6B knockdown (Fig. 5*D*). Knockdown of KDM6B significantly increased the level of H3K27me3, a repressive histone mark that promotes chromatin condensation and can silence HBB expression (34). Concurrently, it suppressed the expression of H3K4me3 and its downstream transcription factor KLF1. These results confirm that KDM6B is a critical mediator in the epigenetic mechanism by which Piezo1 activation promotes HBB production in OA chondrocytes.

**Figure 5:**
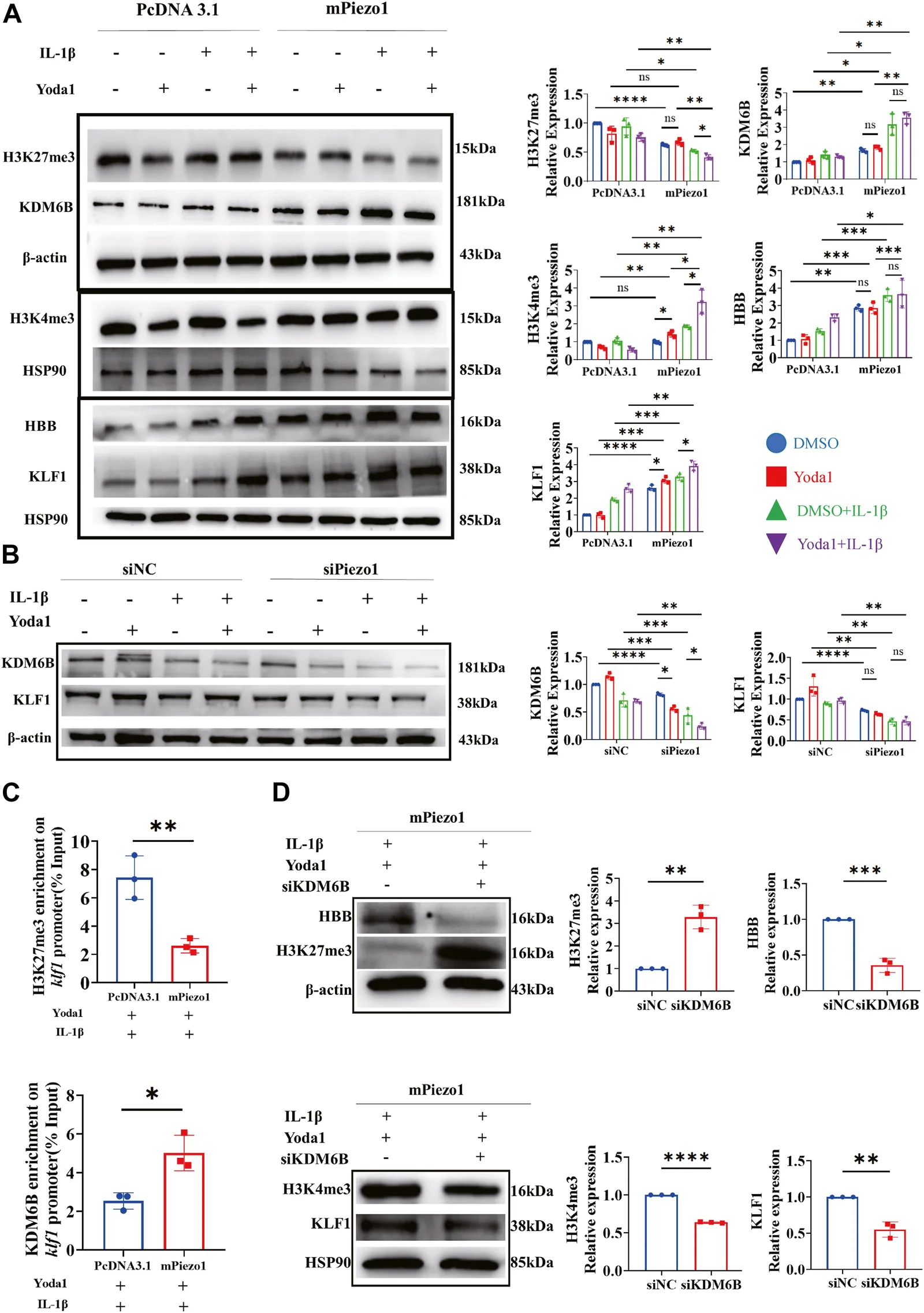
**Piezo1 Regulates HBB via KDM6B/KLF1 Axis.***A*, Representative Western blot bands and quantitative analysis of H3K27me3, KDM6B, HBB, H3K4me3 and KLF1 in the primary chondrocytes transfected with either empty vector (PcDNA3.1-IRES-eGFP) or mPiezo1-overexpression vector after treatment with or without IL-1β and/or Yoda1 for 24 h ns, not significant, ∗*p* < 0.05, ∗∗*p* < 0.01 and ∗∗∗*p* < 0.001, n = 3. *B*, representative Western blot bands and quantitative analysis of KDM6B and KLF1 in the primary chondrocytes transfected with siNC or siPiezo1 in the absence or presence of IL-1β and/or Yoda1 at 24 h ns, not significant, ∗*p* < 0.05, ∗∗*p* < 0.01, ∗∗∗*p* < 0.001 and ∗∗∗∗*p* < 0.0001, n = 3. *C*, ChIP-qPCR analysis was performed to determine the occupancy of H3K27me3 and the enrichment of KDM6B at the KLF1 promoter region in the primary chondrocytes transfected by mPiezo1-overexpression or empty vector in the presence of IL-1β and Yoda1 at 24 h. The recruitment of KDM6B and the occupancy of H3K27me3 at the KLF1 promoter were quantified using specific antibodies against KDM6B and H3K27me3, respectively. Data are presented as percentage of input DNA. ∗*p* < 0.05 and ∗∗*p* < 0.01, n = 3. *D*, representative Western blot bands and quantitative analysis of HBB and H3K27me3 with H3K4me3 and KLF1 in the primary chondrocytes transfected with either empty vector (PcDNA3.1-IRES-eGFP) or mPiezo1-overexpression vector after treatment with or without IL-1β and/or Yoda1 for 24 h ∗∗*p* < 0.01, ∗∗∗*p* < 0.001 and ∗∗∗∗*p* < 0.0001, n = 3.

### Hemoglobin influences energy metabolism and phenotype of Piezo1-mediated OA chondrocytes

Since HBB expression is positively correlated with OA progression, we then investigated if HBB modulates energy metabolism and phenotype of Piezo1-mediated OA chondrocytes. As illustrated in Figure 6, *A* and *B*, under combined Yoda1 and IL-1β treatment for 6 h, HBB knockdown *via* siRNA (Fig. S4*C*) in Piezo1-overexpressing chondrocytes led to a significant decrease in ATP5A1 protein levels, while increasing the expression of LDHA and HIF-1α compared to control cells. At this early time point, there were no significant differences observed in the levels of COL2A1, MMP13, or P16^ink4a^ between the two groups. In contrast, at 24 h post-treatment, HBB knockdown resulted in a significant increase of ATP5A1 expression, accompanied by reduced levels of LDHA and HIF-1α. Furthermore, as shown in Figure 6*C*, HBB knockdown markedly reduced the percentage of SA-β-gal-positive cells, indicating that the inhibition of HBB expression attenuated the senescence phenotype in chondrocytes against the induced cellular senescence. To further characterize the temporal dynamics of HBB-mediated mitochondrial regulation, we performed a time-course analysis of mitoROS production (Fig. 6*D*). In siNC-treated cells, Piezo1 activation induced a transient increase in mitoROS levels, with a peak at 6 h, followed by a decline at later time points. In contrast, siHBB cells showed reduced mitoROS accumulation throughout the stimulation period, indicating that HBB depletion protects OA chondrocytes from excessive mitochondrial oxidative stress (Fig. 6*F*). Concomitantly, TMRM staining revealed that HBB knockdown effectively sustained mitochondrial membrane potential (Fig. 6, *E* and *G*). These findings demonstrate that HBB drives a pathological metabolic cascade in OA chondrocytes, promoting mitochondrial OXPHOS beyond the adaptation capacity of avascular cartilage, thereby leading to ROS-induced mitochondrial dysfunction and a compensatory shift to glycolysis. Notably, HBB knockdown prevents this cascade by maintaining mitochondrial homeostasis and attenuating oxidative stress. To validate the effects of HBB on mitochondrial respiration in OA chondrocytes, we further performed Seahorse extracellular flux analysis. As shown in Figures 7, *A*, *B* and S7, *A*, *B*, at the early induction stage (3 and 6 h), chondrocytes transfected by siHBB exhibited reduced oxidative phosphorylation capacity compared to those transfected by siNC. However, this trend reversed at the late induction stage (12 and 24 h). The siHBB group displayed enhanced oxidative phosphorylation capacity. In terms of glycolysis, the siHBB group showed significantly elevated glycolytic function at the early stage but reduced glycolytic function at the late stage relative to the siNC group. These results demonstrate that HBB silencing effectively preserved mitochondrial respiratory function under Piezo1 activation, maintaining a more stable oxidative phosphorylation capacity and attenuating mitochondrial dysfunction. To further determine whether HBB directly associates with mitochondria under pathological conditions, we performed immunofluorescence staining using an anti-HBB antibody together with MitoTracker (mitochondrial dye). As shown in Figure 7*C*, HBB signals substantially overlapped with MitoTracker-labeled mitochondria in the primary chondrocytes transfected by Piezo1-overexpression vector in the presence of IL-1β and Yoda1 within the entire stimulation period. We propose that this co-localization of HBB and mitochondria facilitates oxidative phosphorylation before the onset of mitochondrial dysfunction driven by excessive ROS accumulation.

**Figure 6:**
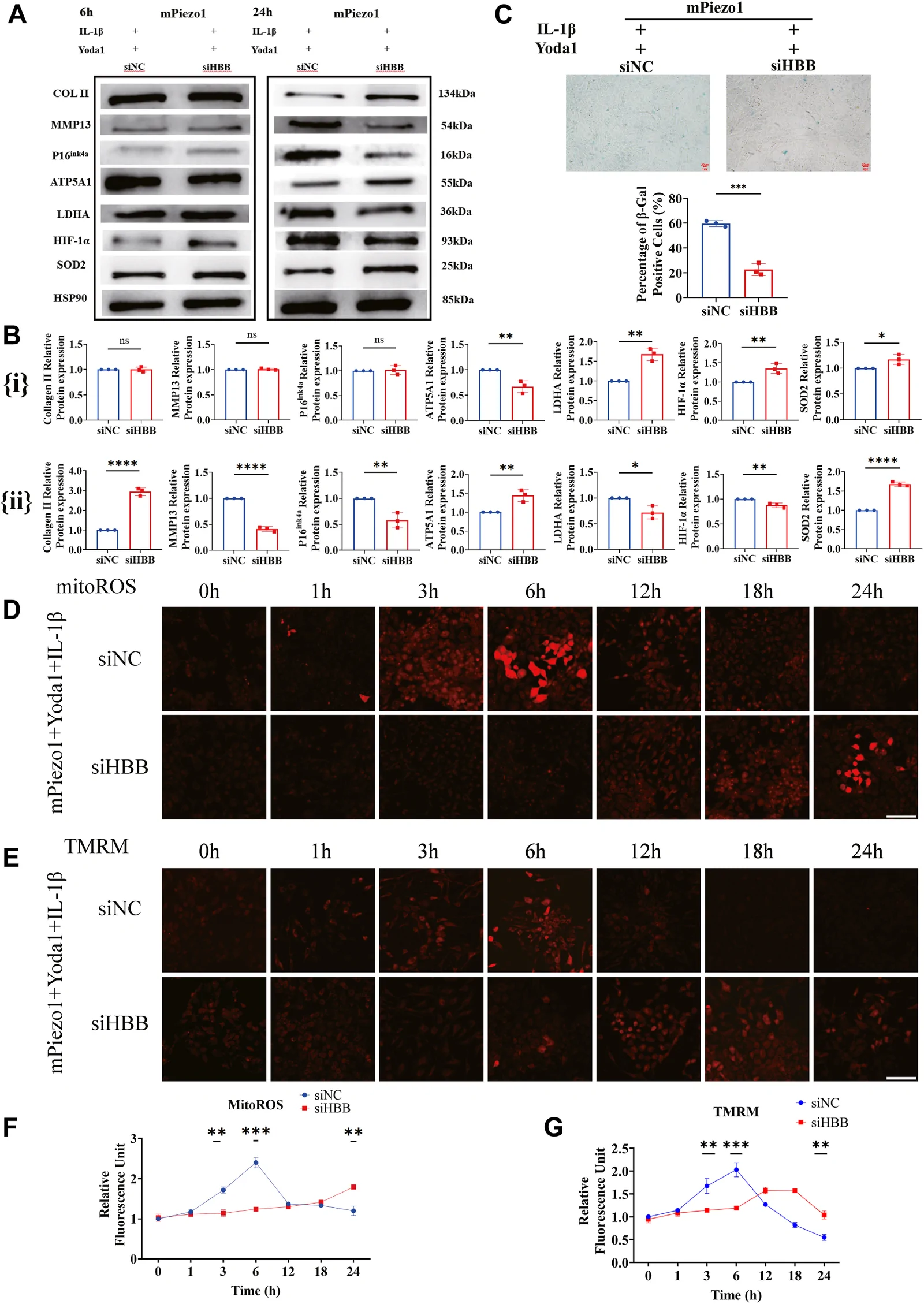
**HBB Knockdown Rescues Mitochondrial Dysfunction.***A*, Representative Western blot bands and quantitative analysis of COL II, MMP13, P16^ink4a^, ATP5A1, LDHA, HIF-1α and SOD2 in the primary chondrocytes transfected by mPiezo1-overexpression vector after treatment with siNC or siHBB in the presence of IL-1β and Yoda1 at 6 and 24 h. *B*, quantitative analysis of COL II, MMP13, P16^ink4a^, ATP5A1, LDHA, HIF-1α, and SOD2 in the primary chondrocytes transfected by mPiezo1-overexpression vector after treatment with siNC or siHBB in the presence of IL-1β and Yoda1 at 6 {i} and 24 {ii} h. ns, not significant, ∗*p* < 0.05, ∗∗*p* < 0.01 and ∗∗∗∗*p* < 0.0001, n = 3. *C*, representative β-Galactosidase Staining of the primary chondrocytes transfected by mPiezo1-overexpression vector after treatment with siNC or siHBB in the presence of IL-1β and Yoda1 at 24 h ∗∗∗*p* < 0.001, n = 3. Scale bar: 20 μm. *D*, representative fluorescence images showing mitochondrial superoxide levels of the primary chondrocytes transfected by mPiezo1-overexpression vector after treatment with siNC or siHBB in the presence of IL-1β and Yoda1 at the indicated time points. Scale bar: 100 μm. *E*, quantification of the relative fluorescence unit in (*D*), ∗*p* < 0.05 and ∗∗∗∗*p* < 0.0001, n = 3. Scale bar: 100 μm. *F*, representative fluorescence images of TMRM staining in the primary chondrocytes transfected by mPiezo1-overexpression vector after treatment with siNC or siHBB in the presence of IL-1β and Yoda1 at the indicated time points. Scale bar: 100 μm. *G*, quantitative analysis of the relative fluorescence unit in (*E*), ∗*p* < 0.05 and ∗∗∗∗*p* < 0.0001, n = 3.

**Figure 7:**
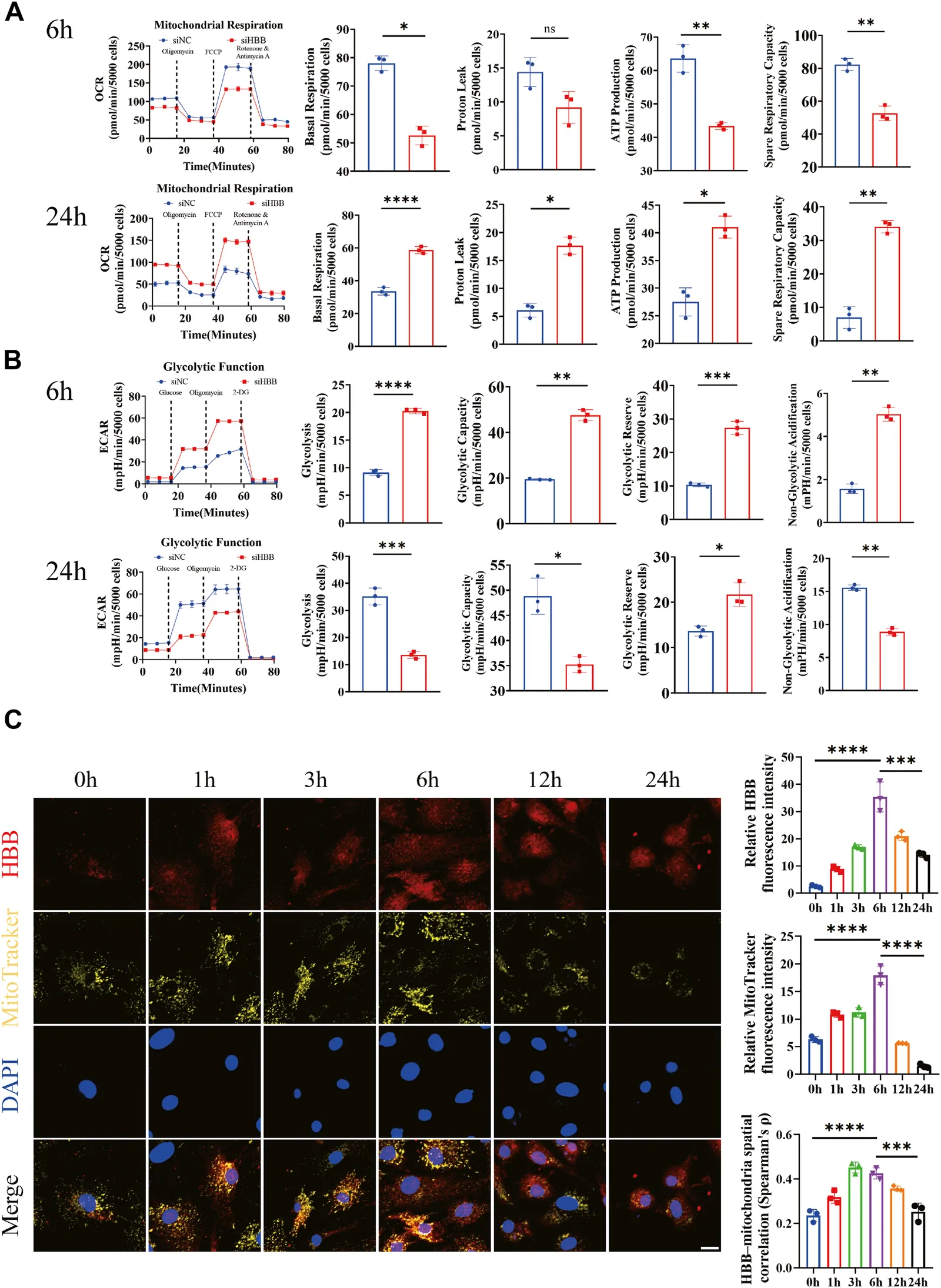
**HBB Modulates Mitochondrial Respiration.***A*, Representative oxygen consumption rate (OCR) profiles and quantitative analysis of mitochondrial respiration parameters, including basal respiration, ATP production, maximal respiration, and spare respiratory capacity in the primary chondrocytes transfected by mPiezo1-overexpression vector after treatment with siNC or siHBB in the presence of IL-1β and Yoda1 at 6 and 24 h ns, not significant, ∗*p* < 0.05, ∗∗*p* < 0.01 and ∗∗∗∗*p* < 0.0001, n = 3. *B*, representative extracellular acidification rate (ECAR) profiles and quantitative analysis of glycolytic parameters, including glycolysis, glycolytic capacity, glycolytic reserve, and non-glycolytic acidification in the primary chondrocytes transfected by mPiezo1-overexpression vector after treatment with siNC or siHBB in the presence of IL-1β and Yoda1 at 6 and 24 h ∗*p* < 0.05,∗∗*p* < 0.01, ∗∗∗*p* < 0.001 and ∗∗∗∗*p* < 0.0001, n = 3. *C*, representative immunofluorescence images showing HBB and mitochondria and quantification analysis of relative fluorescence intensity of HBB and mitochondria, as well as HBB–mitochondria colocalization in the primary chondrocytes transfected by mPiezo1-overexpression vector in the presence of IL-1β and Yoda1 at the indicated time points. Quantification of HBB fluorescence intensity, MitoTracker fluorescence intensity, and HBB–mitochondria spatial correlation (Spearman’s correlation coefficient). ∗∗∗*p* < 0.001 and ∗∗∗∗*p* < 0.0001; n = 3. Scale bar = 20 μm.

### Knockdown of Piezo1 and hemoglobin in chondrocytes attenuated OA progression

As knockdown of either Piezo1 or HBB effectively ameliorated the OA phenotype in chondrocytes, we next evaluated the therapeutic potential of intra-articular (IA) injection of siRNA targeting Piezo1 or HBB (Fig. S6, *B* and *C*), delivered *via* PEI-CAP nanoparticles (NPs) with narrow size distribution (Fig. S8), for attenuating OA progression in mice. siRNA negative control was used as a control. Since the *in vivo* retention time of the siNC conjugated with Cy5.5 dye, loaded in the PEI-CAP NPs, was 1 week after IA injection in mice under *in vivo* imaging system (IVIS) analysis and immunofluorescence detection (Fig. S9), IA administration weekly was conducted in the present study. Furthermore, the biosafety of the PEI-CAP nanoparticles was evaluated by histological examination of major organs, including the heart, liver, spleen, lung, and kidney. No detectable pathological abnormalities were observed, indicating high biocompatibility of the nanoparticle system (Fig. S10). As shown in Figure 8*A*i-ii, siRNA-mediated knockdown of Piezo1 or HBB led to significant improvements in gait performance, including stride length and average print area, at both 4 and 8 weeks after DMM surgery. Concomitantly, the mice with knockdown of either Piezo1 or HBB displayed significant lower OARSI scores and reduced P16^ink4a^ positive cells, alongside elevated COL2A1 expression relative to the control group at both 4 and 8 weeks post-surgery (Fig. 8, *B*–*E*). The results confirmed that knockdown of Piezo1 or HBB in chondrocytes attenuates OA development. In addition, knockdown of either Piezo1 or HBB significantly rescued mitochondrial OXPHOS capability in terms of expression levels of ATP5A1, LDHA, and HIF-1α without causing health risks in mice at both 4 and 8 weeks post-surgery (Fig. S11). Together, these data indicate that Piezo1/HBB axis plays a central role in modulation of energy metabolism of OA chondrocytes and subsequently influences OA pathogenesis.

**Figure 8:**
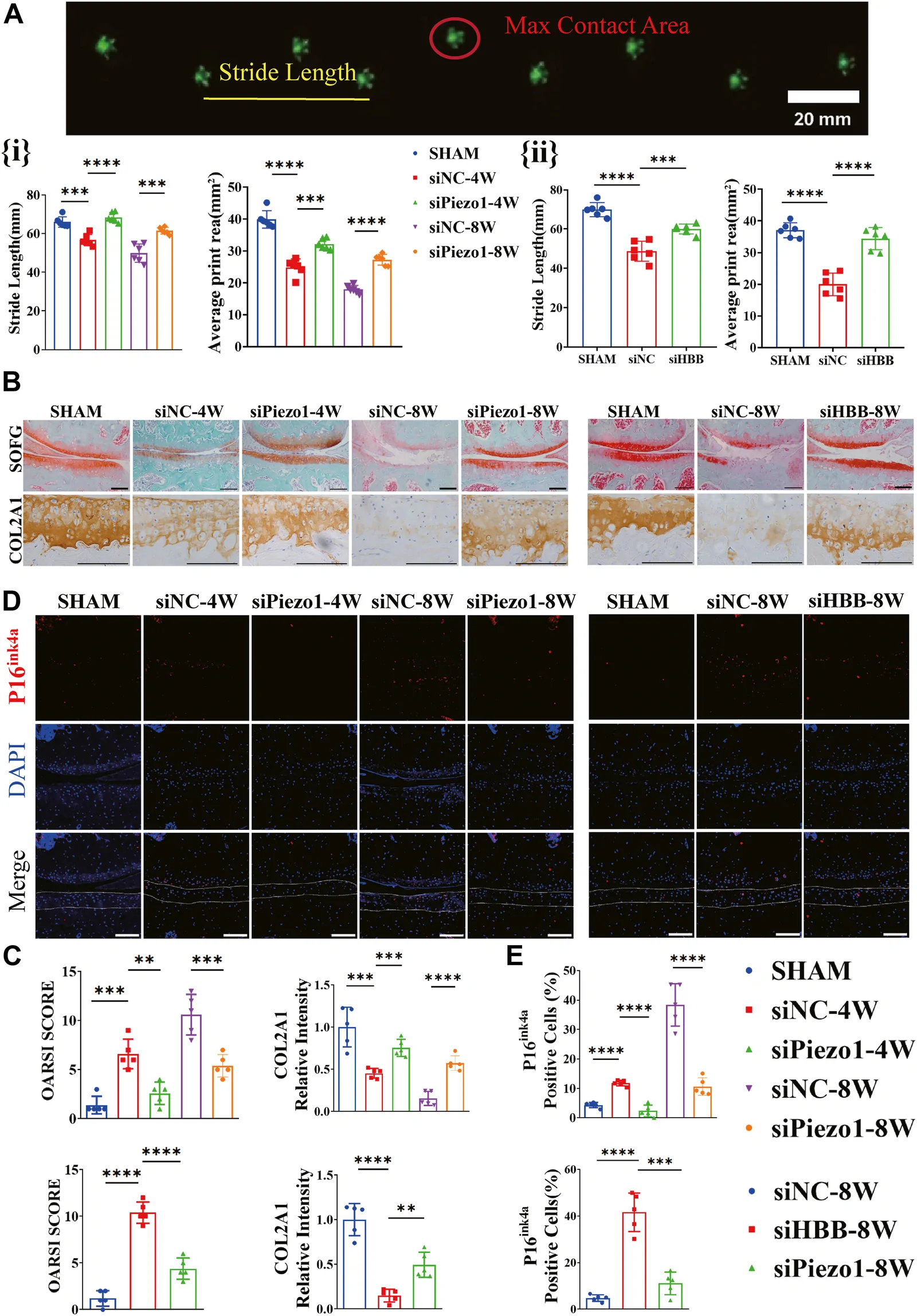
**Piezo1/HBB Knockdown Attenuates OA in Mice.***A,* Schematic illustration of gait analysis and quantitative measurements of stride length and average print area at 4 and 8 weeks post-surgery in mice treated with CAP-PEI loaded with siNC or siPiezo1 {i}, and at 8 weeks in mice treated with CAP-PEI loaded with siNC or siHBB {ii}, with Sham-operated mice as controls in both series. ∗*p* < 0.05, ∗∗*p* < 0.01, and ∗∗∗*p* < 0.001, n = 6. Scale bar: 20 mm. *B*, representative histological (Safranin O staining) and immunohistochemistry images of knee articular cartilage at 4 and 8 weeks post-surgery in mice treated with CAP-PEI loaded with siNC or siPiezo1, and at 8 weeks in mice treated with CAP-PEI loaded with siNC or siHBB, with sham-operated mice as controls in both series. Scale bar: 100 μm. *C*, quantification of the indicated OARSI score and COL2A1 relative intensity in (*B*). ∗∗*p* < 0.01, ∗∗∗*p* < 0.001 and ∗∗∗∗*p* < 0.0001, n = 5. Scale bar: 100 μm. *D*, representative immunofluorescence images of knee articular cartilage showing P16^ink4a^ expression at 4 and 8 weeks post-surgery in mice treated with CAP-PEI loaded with siNC or siPiezo1, and at 8 weeks in mice treated with CAP-PEI loaded with siNC or siHBB, with sham-operated mice as controls in both series. Scale bar: 100 μm. *E*, quantitative analysis of P16^ink4a^ positive cells at the articular cartilage. ∗∗∗*p* < 0.001 and ∗∗∗∗*p* < 0.001, n = 5. Scale bar: 100 μm.

## Discussion

The present study delineates the underlying mechanism behind the effects of Piezo1 on trauma or obesity-induced OA progression. Our findings demonstrate that the abnormal distribution of mechanical stress in articular cartilage causes aberrant increase of Piezo1 expression, thereby resulting in elevated level of HBB *via* activation of KDM6B/KLF1 signaling pathway. Enhanced HBB expression initially facilitates mitochondrial OXPHOS and subsequently causes ROS-induced mitochondrial dysfunction, ultimately leading to chondrocyte senescence. Of note, either Piezo1 or HBB knockdown significantly restores mitochondrial OXPHOS capability and then ameliorates OA progression (Fig. 9).

**Figure 9:**
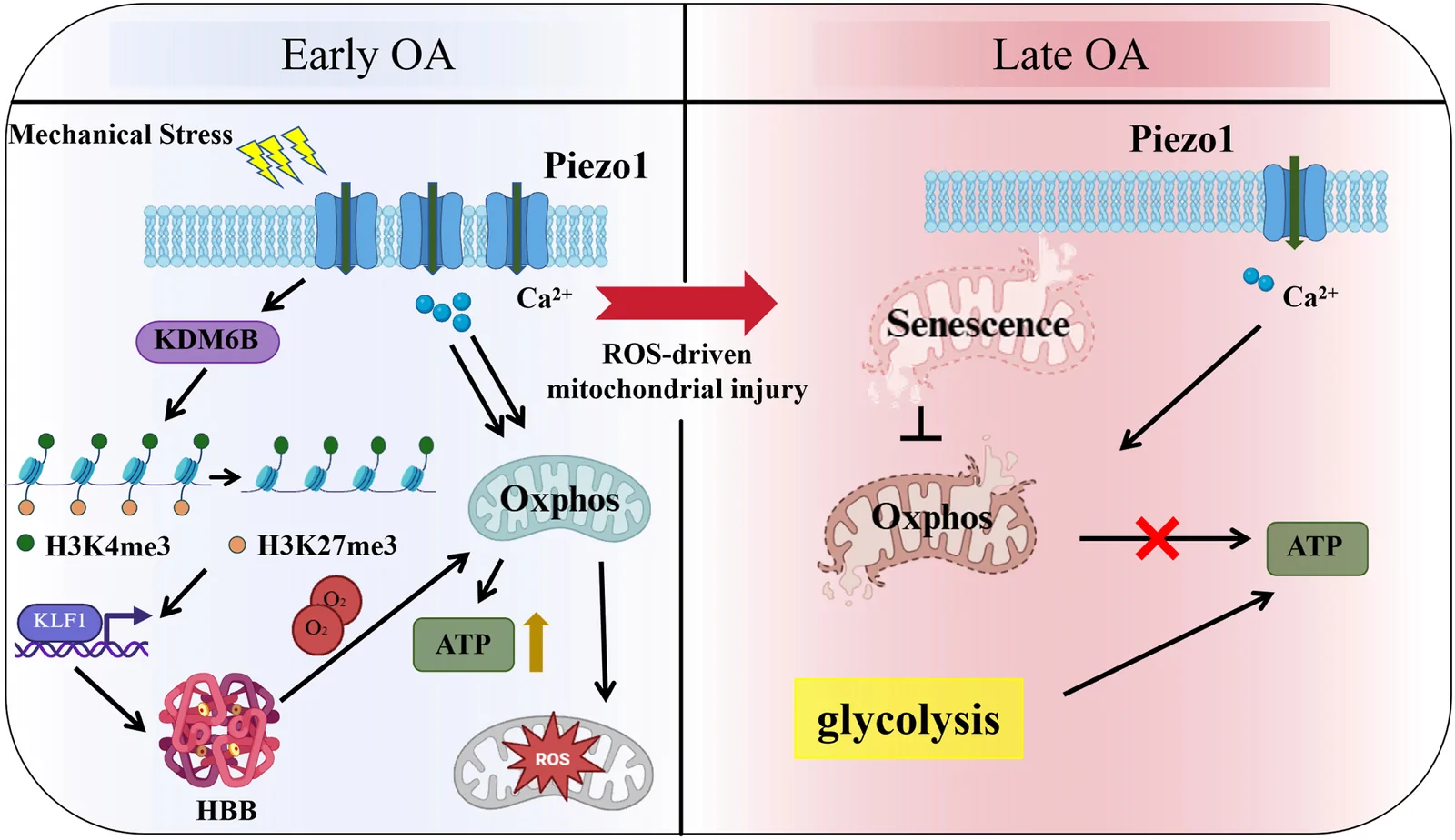
**Schematic diagram showing the underlying mechanism behind the effects of Piezo1-mediated HBB upregulation on OA progression caused by aberrant mechanical stress.** Overload stimulates Piezo1 overexpression in chondrocytes, thereby improving HBB production through activation of KDM6B/KLF1 signaling pathway. Consequently, it initially leads to enhanced mitochondrial OXPHOS in OA chondrocytes and subsequently results in cellular senescence due to excessive ROS accumulation, which contributes to the energy metabolism shift from mitochondrial OXPHOS to glycolysis in chondrocytes at the advanced OA stage.

Emerging evidence demonstrates that Piezo1, a mechanosensitive ion channel, serves as a critical regulator exacerbating OA progression (6, 35). Sun *et al.* reported that mechanical stress results in a profound increase of Piezo1 expression and subsequent mitochondrial Ca^2+^ overload, thereby causing promoted release of mitochondrial DNA and cell senescence (20). Of note, on the one hand, Piezo1 activation improves the activity of Hexokinase 2 and in turn promotes glycolysis (7), on the other hand, Piezo1 activation increases the mitochondrial oxygen consumption rate during enhanced OXPHOS (24). Based on these facts, we raised a hypothesis that OA chondrocytes, with aberrant activation of Piezo1, display initially improved mitochondrial OXPHOS and subsequently shift their primary energy reliance to glycolysis to meet metabolic demands due to ROS-induced mitochondrial dysfunction. However, it still remains unclear how Piezo1 modulates mitochondrial OXPHOS and thereby exacerbates OA progression. In the present study, for the first time, we reported that hemoglobin, previously as the oxygen carrier to maintain chondrocyte homeostasis (12), is also a critical regulator orchestrating energy metabolism in response to Piezo1 activation in OA chondrocytes. Importantly, our data revealed that Piezo1 expression showed an aberrant increase initially in the articular cartilage of mice with OA models induced by DMM, ACLT, or obesity, but not in those with age-related OA, indicating that Piezo1 may not be the primary cause inducing OA pathogenesis in an aging population. Indeed, accumulating evidence reports that chondrocyte senescence with age and alterations in cell signaling may be the major factors related with age-induced OA (23). Of note, the upregulation of Piezo1 coincided with an increase in HBB level, but not HBA. Concurrently, we observed a significant elevation in ATP5A1 expression in articular cartilage during the early stage of OA. However, as OA progressed, ATP5A1 expression decreased abruptly, while the levels of LDHA and HIF-1α were markedly increased. These findings suggest that Piezo1-mediated HBB upregulation is accompanied with enhanced mitochondrial OXPHOS, which may in turn lead to excessive ROS production and subsequent mitochondrial dysfunction.

To further elucidate the role of HBB in OA phenotype and energy metabolism in chondrocytes, siRNA-mediated knockdown of HBB was conducted. Notably, in Piezo1-overexpressing OA chondrocytes, knockdown of HBB effectively suppressed ROS accumulation and sustained mitochondrial function, an effect attributable to the suppression of the initial surge in mitochondrial OXPHOS. Furthermore, *in vitro* data also confirmed that overexpression of Piezo1 in OA chondrocytes resulted in remarkable upregulation of HBB. Compared to the Piezo1-overexpressing OA chondrocytes with HBB knockdown, the Piezo1-overexpressing OA chondrocytes displayed significantly higher level of ATP5A1 while lower expression of LDHA and HIF-1α at 6 h post-treatment. In contrast, improved mitochondrial OXPHOS results in ROS overproduction in OA chondrocytes. As excessive ROS may impair mitochondrial function, we then observed a significant lower expression of ATP5A1 while higher levels of LDHA and HIF-1α at 24 h post-treatment in the Piezo1-overexpressing OA chondrocytes relative to the cells with HBB knockdown. Importantly, in chondrocytes overexpressing Piezo1, its activation and pro-inflammatory cytokines act synergistically to induce the expression of HBB. In sharp contrast, these pro-inflammatory cytokines alone failed to alter HBB levels in chondrocytes expressing Piezo1 at baseline levels, underscoring that Piezo1 overexpression is a prerequisite for the inflammatory upregulation of HBB. Consistently, the age-related OA leads to high expression of inflammatory cytokines, *for example*, senescence-associated secretory phenotype (SASP) in chondrocytes (23, 36), but there was no aberrant increase of Piezo1 level in articular cartilage of the age-related OA mice, indicating that HBB is not the factor contributing to OA progression in the aging population. This is further evidenced by HBB expression results in articular cartilage of the aged mice (Fig. S5). Interestingly, despite a decrease in Piezo1 expression at the advanced OA stage in both DMM and ACLT mouse models, the proportion of HBB-positive chondrocytes exhibited a persistent increase within the articular cartilage, which may be ascribed to vascular invasion into the calcified cartilage (37).

In addition, our findings provide compelling evidence that Piezo1 activation upregulates HBB production through activating the KDM6B/KLF1 signaling pathway in OA chondrocytes, which is negatively correlated with H3K27me3 expression but positively correlated with H3K4me3 enrichment. The delineation of the underlying mechanism associated with Piezo1-mediated production of HBB may provide the potential therapeutic targets of trauma or obesity-induced OA. Under physiological condition, KDM6B acts as a critical histone demethylase to support cartilage development, and its deficiency may lead to impaired cartilage formation and accelerated OA progression (38). However, in pathological condition, pharmacological inhibition of KDM6B has emerged as an effective strategy to attenuate OA development by prevention of H3K27me3 loss, thereby suppressing cartilage damage in the experimental OA model (39). Beyond KDM6B, KLF transcription factors also participate in the pathological process of bones and joints (40), which aligns with our findings in the present study. Importantly, Piezo1 and HBB, as the upstream and the downstream component in the Piezo1-mediated signaling pathway, respectively, were also identified as the potential therapeutic targets for OA intervention. Consistently, knockdown of either Piezo1 or HBB significantly restored mitochondrial OXPHOS capability, which may be ascribed to suppression of ROS-induced mitochondrial dysfunction, ultimately leading to the attenuation of OA progression.

In summary, Piezo1 exhibits an aberrant increase in articular cartilage of trauma and obesity-induced OA, thereby leading to improved expression of HBB *via* activation of KDM6B/KLF1 signaling pathway and initial enhancement of mitochondrial OXPHOS. The overproduction of ROS subsequently impairs mitochondrial function and consequently results in energy metabolism shift from mitochondrial OXPHOS to glycolysis at the advanced OA stage. Importantly, Piezo1 works synergistically with pro-inflammatory cytokines to induce HBB expression and drive OA progression. The present work elucidates the underlying mechanism behind the effects of Piezo1-mediated HBB on OA pathogenesis and identifies both Piezo1 and HBB as potential therapeutic targets for mitigating mechanical stress-induced OA.

## Experimental procedures

### Establishment of animal models

All animal experiments were approved by the Institutional Animal Care and Use Committee of Sun Yat-sen University (Approval No.2024001967). All male mice in this study were on a C57BL/6 background and were purchased from Zhuhai Bestest Biotechnology Co., Ltd. Animals were included in the study only if they met the following conditions: 8 weeks old, weighed within 20 to 25 g, exhibited normal grooming and exploratory behavior during a 7-days acclimation period. Mice were housed under specific pathogen-free (SPF) conditions at 22 ± 2 °C with 50 to 60% humidity on a 12-h light/dark cycle, with free access to food and water. Animals were randomly assigned to cages upon arrival. Furthermore, cage positions on the rack were systematically rotated every week to minimize any potential confounding effects of environmental gradients. All measurements and statistical analyses were performed with the individual mouse as the experimental unit. Allocation of experimental units to the model and control groups was based on a computer-generated randomization sequence.

#### DMM/ACLT surgery

Eight-week-old wild male C57BL/6J mice were placed in a supine position with the left hind limb fixed and the knee flexed at 90°. After intraperitoneal anesthesia, the skin at the surgical area was thoroughly disinfected with iodophor. After that, an incision was created in the skin to expose the patellar tendon prior to dislocation of the patella through making the incision along the medial edge of the patellar tendon using a scalpel. Intra-articular adipose tissue was bluntly dissected and removed. The medial meniscotibial ligament (MMTL) or the anterior cruciate ligament (ACL) was transected to establish DMM or ACLT model. The wound was irrigated, and the joint cavity and skin incision were sutured by layers. The exposure of knee joint cavity was only conducted in the SHAM surgery group. In the DMM model, the mice were randomly divided into four groups (n = 5 per group): three groups underwent DMM surgery and were euthanized at 2, 4, or 8 weeks post-surgery, respectively, and one SHAM-operated group served as control. Similarly, in the ACLT model, the mice were randomly divided into four groups (n = 5 per group): three groups underwent ACLT surgery and were euthanized at 1, 3, or 6 weeks post-surgery, respectively, and one SHAM-operated group served as control. At each endpoint, knee joints were harvested for subsequent analysis.

#### High-fat diet induced obesity with wheel-running exercise (HFR)

Four-week-old C57BL/6 wild male mice were fed a high-fat diet purchased from Guangdong Medical Laboratory Animal Center. After 12 weeks of feeding, obesity was considered successfully modeled if the body mass of the animals was 20% or more above the mean body mass of the control group (control group mean weight: 28.455 ± 2.175 g; obesity model group mean weight: 38.565 ± 1.735 g). The obese mice were then subjected to running exercise using a small animal running wheel system (SA103, Sanbio, Jiangsu, China) (Fig. S12). The running protocol consisted of 80 min per session at a speed of 15 m/min, 0° incline, 5 sessions per week. In the obesity-induced model, the mice were randomly divided into four groups (n = 5 per group): three groups underwent HFR treatment and were euthanized at 2, 4, or 8 weeks after exercise, respectively, and the healthy mice without high-fat diet and wheel-running exercise were set as control.

#### Age-related OA mouse model

Wild male C57BL/6 mice aged 6, 8, 10, 12, 19, and 24 months (n = 3 per group) were directly used for subsequent experiments.

### Histological analysis

The decalcified tissues were embedded in paraffin, sectioned and stained with safranin O/fast green (Roles-Bio, Catalog: BS10017) prior to histological assessment using the OARSI grading system (Table S1). For immunohistochemistry, sections were incubated with primary antibodies COL II (diluted 1:2000, Proteintech, Catalog: 28459-1-AP) at 4 °C overnight and HRP-labeled secondary antibodies (Servicebio, Catalog: G1302) for 30 min at room temperature, and then stained with DAB peroxidase substrate kit (Solarbio, Catalog: DA1016). For immunofluorescence staining, the primary antibodies used were as follows: Piezo1 (diluted 1:1000, Proteintech, Catalog: 28511-1-AP), p16^ink4a^ (diluted 1:1000, Affinity, Catalog: AF5484), LDHA (diluted 1:2000, Proteintech, Catalog: 19987-1-AP), HIF-1α (diluted 1:2000, Proteintech, Catalog: 20960-1-AP), HBB (diluted 1:500, Proteintech, Catalog: 16216-1-AP) and ATP5A1 (diluted 1:2000, Proteintech, Catalog: 14676-1-AP). Afterwards, incubation with the AF488/AF647 goat anti-rabbit IgG H&L secondary antibody (diluted 1:1000, Abcam, Catalog: 28459-1-AP) was conducted for 1 h at room temperature, along with nuclei staining with DAPI. Images were acquired by confocal microscope (AX/AXR, Nikon).

### Cell culture and transfection

To preserve the native metabolic phenotype of primary chondrocytes, subsequent experiments were conducted using passage 0 (P0) cells. Primary chondrocytes were isolated from the knee joint cartilage of 1-week-old male C57BL/6 mice. The collected chondrocytes were placed in Dulbecco's Modified Eagle Medium/Nutrient Mixture F-12 (DMEM/F12, Gibco) with 10% Fetal Bovine Serum (Excell, Catalog: FSP500), 100 IU/ml penicillin and 100 μg/ml streptomycin (Gibco, Catalog: 15140122) for culture in an incubator at 37 °C with 5% CO_2_. Lipofectamine LTX and PLUS Reagents (Invitrogen, Catalog: 15338100) were used for plasmid transfection in the chondrocytes. Transfection was performed when the chondrocytes reached approximately 50% to 80% confluence. Taking a 6-well plate as an example, the reagents per well were: 2.5 μg plasmid, 2.5 μl PLUS Reagent, 5 μl Lipofectamine LTX, and 125 μl reduced-serum medium (OPTI-MEM, Thermo Fisher). siRNA transfection can be performed 24 h after plasmid transfection. Taking a 6-well plate as an example: for each well, dilute 55 pmoles of target siRNA (siHbb: 5′-UGATAACTGCCTTTAACGA-3′, sense and 5′-UCGUUAAAGGCAGUUAUCA-3′, antisense; siKdm6b: 5′-CCCUAACAAACCCUAUUAU-3′, sense 5′-AUAAUAGGGUUUGUUAGGG-3′, antisense) in 125 μl OPTI-MEM and 4 μl of PEI 40000 (APExBIO, Catalog: K1029) transfection reagent and mix. The Piezo1 agonist Yoda1 (MCE, Catalog: HY-18723) and IL-1β (Novoprotein, Catalog: C042) were used to treat the transfected cells at final concentrations of 10 μM Yoda1 and 10 ng/ml IL-1β.

### Chromatin immunoprecipitation (ChIP)-qPCR

ChIP assays were performed using the BeyoChIP ChIP Assay Kit (Beyotime) according to the manufacturer’s instructions to determine the enrichment of KDM6B and the histone modification marker H3K27me3 at the Klf1 promoter region.

Briefly, the P0 primary chondrocytes transfected with the mouse Piezo1 over-expression plasmid were treated with IL-1β and Yoda1 for 24 h. Cells were harvested and cross-linked with formaldehyde to preserve DNA–protein interactions. Following chromatin preparation and fragmentation, the sheared chromatin was immunoprecipitated with specific antibodies against KDM6B (Abmart, Catalog: o15054) and H3K27me3 (Affinty, Catalog: DF6941), with normal IgG serving as a negative control. The immunoprecipitated DNA was purified according to the kit protocol and subjected to quantitative PCR analysis.

ChIP-qPCR was performed using primers targeting the promoter region of mouse Klf1. The primer sequences are listed in Table S2. The enrichment of KDM6B and H3K27me3 at the Klf1 promoter region was calculated as the percentage of input DNA (% input).

### Quantitative real-time polymerase chain reaction (qRT-PCR) analysis

Relative mRNA expression levels were calculated using the 2^−ΔΔCt^ method with GAPDH (or β-actin) as the internal reference gene. The primers were designed by Shanghai Sangon, and sequences are listed in Table S2.

### Western blotting

Proteins extracted from chondrocytes were separated by SDS-PAGE and transferred to activated PVDF membranes. The membranes were blotted with primary antibodies recognizing LDHA, p16^ink4a^, ATP5A1, HIF-1α, HSP90 (diluted 1:20,000, Proteintech, Catalog: 60318-1-Ig), β-actin (diluted 1:50,000, Proteintech, Catalog: 66009-1-Ig), HBB, MMP13 (diluted 1:2000, Proteintech, Catalog: 18165-1-AP), SOD2 (diluted 1:20,000, Proteintech, Catalog: 24127-1-AP) and Collagen Type II. All blots were probed with horseradish peroxidase (HRP)-conjugated secondary antibodies (ZEN-BIOSCIENCE, Catalog: 550094) and visualized using an enhanced chemiluminescence (ECL) detection system (Tanon 5200, Tanon).

### Senescence-associated β-galactosidase (SA-β-gal) staining

All procedures were performed according to the manufacturer's instructions provided with the senescence β-galactosidase staining kit (Solarbio, Catalog: G1580). After staining, cells were examined under a light microscope (BDS400, Chongqing Optec Instrument Co., Ltd).

### mitoROS staining

MitoSOX Red mitochondrial superoxide indicator (ThermoFisher, Catalog: M36008) specifically targeted to mitochondria in live cells. Oxidation of the MitoSOX reagent by mitochondrial superoxide produces bright red fluorescence. The images were captured under confocal microscope (AX/AXR, Nikon Japan).

### TMRM staining

Tetramethylrhodamine methyl ester (Solarbio, Catalog: IT3860) is a cationic fluorescent probe whose detection principle is based on a specific response to the mitochondrial electrochemical gradient. The images were captured under confocal microscope (AX/AXR, Nikon Japan).

### Cell immunofluorescence staining

Mitochondria were labeled using MitoTracker mitochondrial fluorescent probe (ThermoFisher, Catalog: M7510) according to the manufacturer's instructions. After blocking, cells were incubated overnight at 4 °C with primary antibody against HBB. Subsequently, cells were incubated with appropriate fluorescent secondary antibodies for 1 h at room temperature. The images were captured under confocal microscope (AX/AXR, Nikon).

### Nanoparticles (NP) synthesis and characterization

siPiezo1, sense 5′-ACCAAGAAAUACAACCAUCUA-3′ and antisense 5′-UAGAUGGUUGUAUUUCUUGGU-3′; siHBB, sense 5′-UGATAACTGCCTTTAACGA-3′ and antisense 5′-UCGUUAAAGGCAGUUAUCA-3′. All siRNAs and a negative control siRNA were purchased from Shanghai Sangon. Cy5.5-labeled negative control siRNA were purchased for fluorescent tracking during the *in vivo* experiments.

5 mg of PEI (25 kDa, Sigma-Aldrich, Catalog: 408727) was dissolved in 500 μl of dimethyl sulfoxide (Sigma-Aldrich, Catalog: D8418) to 10 mg mL^−1^ concentration. Approximately 0.5 mg of m-maleimidobenzoyl-N-hydroxysuccinimide ester (Sigma-Aldrich, Catalog: M2786) was added to the PEI solution, which was incubated for 15 min at 37 °C to activate the PEI 5 mg peptides (DWRVIIPPRPSAC, CAP) were dissolved in 500 μl of ultra-pure water. The mixture was added dropwise to the activated PEI, mixed repeatedly, and incubated overnight at 4 °C for conjugation. The pep-PEI solution was purified using a 36 mm Amresco dialysis membrane with 8000 to 15,000 MW cutoff (Viskase) against ultra-pure water for more than 24 h. The water was replaced at least five times. The pep-PEI was lyophilized and stored at −20 °C. The pep-PEI was then dissolved in water to 10 mg mL^−1^ concentration. The siRNAs were diluted with Tris-EDTA buffer (Tris-hydrochloride buffer, pH 8.0, containing 1.0 mM EDTA) to 80 μg/100 μl concentration.

### Gait analysis

The gait analysis for the mice after intra-articular injection with hydrogels was investigated by means of an automated Animal Gait analysis system (Runway Scan, CleverSys Inc.). Prior to the gait recording, the mice were placed freely on a glass platform to walk for 10 min twice per day, which lasted for 5 days to achieve a brief training session. Then, each mouse was placed individually in the walkway and walked freely from one side to the other side while the gait changes were recorded and analyzed by the software.

### Statistical analysis

Details concerning sample sizes, biological replicates, and statistical methodologies are described in the corresponding Figure captions. All *in vitro* experiments in the main text were performed at least three times. In terms of *in vivo* experiments, mice were randomly divided into control and treatment groups, and observers performing surgery and analysis were blinded whether the samples were from control or treatment groups. Experimental data are presented as mean ± standard deviation (x¯ ± SD). Statistical analysis was performed using SPSS statistical software (version 22.0), and graphs were generated using GraphPad Prism 10. All quantitative experiments were conducted with at least three biological replicates. In terms of parametric tests, comparisons between two groups were analyzed using an independent samples *t* test, while multi-group data were analyzed using one-way analysis of variance (ANOVA) with Bonferroni's multiple comparisons correction. The Kruskal–Wallis test was used for multiple group comparisons of nonparametric data, such as OARSI scores. Statistical significance was denoted using an asterisk system: ∗*p* < 0.05, ∗∗*p* < 0.01, ∗∗∗*p* < 0.001, ∗∗∗∗*p* < 0.0001 while ns is considered not significant. Sample size calculation in mice was performed based on the primary outcome (OARSI score) using G∗Power software, with type I error (α) and power (1-β) set to 0.05 and 0.8, respectively.

## Data and materials availability

All data needed to evaluate the conclusions in the paper are present in the paper and/or the Supplementary Materials.

## Supporting information

This article contains supporting information.

## Conflict of interest

The authors declare that they have no conflicts of interest with the contents of this article.
