# Supplementary material for: Piezo1 accelerates osteoarthritis progression via promotion of HBB-dependent oxidative phosphorylation
Source: J Biol Chem. 2026 Aug 25;302(10):113489. doi: 10.1016/j.jbc.2026.113489 (PMC13629211; doi:10.1016/j.jbc.2026.113489)
Supplement: Supplementary file 1 [file mmc1.docx]

**Supplementary Figures**

**Fig.S1.** (A) Representative histological (Safranin O staining) and immunohistochemistry images showing the knee articular cartilage at 2, 4 ,8 weeks in HFR mouse models, 1, 3, 6 weeks in ACLT mouse models, with sham-operated/Ctrl mice as controls in both series and quantitative analysis of OA progression using the OARSI score and COL2A1 relative expression. *P<0.05, **P<0.01, ***P<0.001 and ****P < 0.0001, n = 5. Scale bar: 100 μm. (B) Representative immunofluorescence images showing Piezo1/P16^ink4a^ expression at the knee articular cartilage at 2, 4 ,8 weeks in HFR mouse models, 1, 3, 6 weeks in ACLT mouse models, with sham-operated/Ctrl mice as controls in both series, , n = 5. Scale bar: 100 μm. (C) Quantitative analysis of Piezo1/P16^ink4a^ positive cells at the articular cartilage. ns: not significant, *P<0.05, **P<0.01, ***P<0.001 and ****P < 0.0001, n = 4. (D) mRNA (qPCR) levels of *Il-1β* and *Mmp13* of the knee articular cartilage at 2, 4 ,8 weeks in HFR mouse models, 1, 3, 6 weeks in ACLT mouse models, with sham-operated/Ctrl mice as controls in both series. ns: not significant, *P<0.05, **P<0.01, ***P<0.001 and ****P < 0.0001, n = 3.

**Fig. S2.** Scatter plots compare mechanical instability-induced OA models, including DMM (2 and 4 weeks), ACLT (1 and 3 weeks), and obesity-associated mechanical overload OA (HFR; 2 and 4 weeks), along with their corresponding sham/control groups, against age-related OA mice (6, 8, 10, and 12 months). Each point represents the Piezo1-positive cell rate (%) and the corresponding OARSI score for an individual animal. Spearman’s rank correlation was used to evaluate the relationship between Piezo1 abundance and cartilage degeneration severity. Significant positive correlations were observed in DMM, ACLT, and HFR OA models, but not in aging-associated OA model. These data demonstrate that Piezo1 upregulation correlates with mechanical overload rather than general OA severity.

**Fig.S3.** Representative western blot bands and quantitative analysis of mitochondrial ATP5A1, glycolytic LDHA and hypoxia marker HIF-1α in knee articular cartilage from DMM-operated mice at 2, 4, and 8 weeks post-surgery. The uncropped full-length membrane images, pre-exposure while-light images, and corresponding ECL chemiluminescence images are presented. Cropped regions used for quantification are indicated on the full-length membranes. These images provide validation of antibody specificity, membrane integrity, and original signal acquisition.

**Fig. S4.** (A) Representative immunofluorescence images showing eGFP expression in the primary chondrocytes transfected with either mPiezo1-overexpression plasmid or vector control (PcDNA3.1-IRES-eGFP). Scale bar: 100 μm. (B) Representative western blot bands of Piezo1 in the primary chondrocytes transfected with either mPiezo1-overexpression plasmid or vector control (PcDNA3.1-IRES-eGFP). (C) Representative western blot bands of HBB in the primary chondrocytes transfected with either mPiezo1-overexpression plasmid or vector control (PcDNA3.1-IRES-eGFP) at 24 h. (D) Representative western blot bands of KDM6B in the primary chondrocytes transfected with mPiezo1-overexpression plasmid after treatment with siNC or siKDM6B in the presence of IL-1β and Yoda1 at 24 h. (E) Agarose gel electrophoresis confirmed the specific amplification of the KLF1 promoter fragment from ChIP samples using antibodies against KDM6B and H3K27me3, with input DNA as control.

**Fig.S5.** Representative immunofluorescence images and quantitative analysis of HBB expression at the knee articular cartilage of mice at indicated age ranging from 6-24 months. The outline of tibial cartilage is indicated by a white dashed dotted line. ns: not significant , n = 3. Scale bar: 100 μm.

**Fig.S6.** (A) Representative western blot bands of Piezo1 and HBB in the primary chondrocytes transfected by Piezo1 overexpression vector after treatment with siNC or siPiezo1 in the presence of IL-1β and Yoda1 at 24 h. (B) Representative immunofluorescence images showing Piezo1 and HBB expression at knee articular cartilage in mice treated with CAP-PEI loaded with siNC or siPiezo1 at 4 and 8 weeks post-surgery and with CAP-PEI loaded siNC or siHBB at 8 weeks post-surgery. Scale bar: 100 μm. (C) Quantitative analysis of Piezo1/HBB positive cells at the articular cartilage. ***P<0.001 and ****P<0.001, n = 5.

**Fig.S7.** (A) Representative oxygen consumption rate (OCR) profiles and quantitative analysis of mitochondrial respiration parameters, including basal respiration, ATP production, maximal respiration, and spare respiratory capacity in the primary chondrocytes transfected by mPiezo1-IRES-eGFP after treatment with siNC or siHBB in the presence of IL-1β and Yoda1 at 3 and 12 h. ns: not significant, *P<0.05, **P<0.01 and ***P<0.001, n = 3. (B) Representative extracellular acidification rate (ECAR) profiles and quantitative analysis of glycolytic parameters, including glycolysis, glycolytic capacity, glycolytic reserve, and non-glycolytic acidification in the primary chondrocytes transfected by mPiezo1-IRES-eGFP after treatment with siNC or siHBB in the presence of IL-1β and Yoda1 at 3 and 12 h. *P<0.05,**P<0.01, ***P<0.001 and ****P < 0.0001, n = 3.

**Fig.S8.** (A) Loading capacity and encapsulation efficiency of CAP-PEI/siRNA complexes including CAP-PEI/siNC nanoparticles, CAP-PEI/siHBB nanoparticles, and CAP-PEI/siPiezo1 nanoparticles (B) Zeta potential of the CAP-PEI/siRNA complexes. (C) Size distribution of the CAP-PEI/siRNA complexes.

**Fig.S9.** (A) In vivo fluorescence images of mice at day0, day1, day2, day3, day5, and day7 after intra-articular injection of CAP-PEI/siNC-Cy5.5. (B) Representative immunofluorescence images of Cy5.5 expression at the knee articular cartilage at the indicated time points after intra-articular injection of CAP-PEI/siNC-Cy5.5. Scale bar: 100 μm.

**Fig. S10**. Representative histological images (H&E) of heart, liver, spleen, lung, and kidney from mice at 8 weeks after intra-articular injection of CAP-PEI/siNC (siNC-NPs), CAP-PEI/siHBB(siHBB-NPs), and CAP-PEI/siPiezo1(siPiezo1-NPs). Scale bars: 100 μm.

**Fig.S11**. (A) Representative immunofluorescence images of knee articular cartilage showing ATP5A1, LDHA and HIF-1α expression at 4 and 8 weeks post-surgery in mice treated with CAP-PEI loaded with siNC or siPiezo1, and at 8 weeks in mice treated with CAP-PEI loaded with siNC or siHBB, with sham-operated mice as controls in both series. Scale bar: 100 μm. (B) Quantitative analysis of ATP5A1, LDHA and HIF-1α positive cells at the articular cartilage. *P<0.05, **P<0.01,***P<0.001 and ****P<0.001, n = 5. Scale bar: 100 μm.

**Fig.S12.** (A) Schematic diagram of the experimental design for high-fat diet-induced obesity with wheel-running exercise (HFR) OA mice model. (B) Images of the in-cage small animal running wheel system (SA103, Sanbio, Jiangsu, China).
