# Supplementary material for: Piezo1 accelerates osteoarthritis progression via promotion of HBB-dependent oxidative phosphorylation
Source: J Biol Chem. 2026 Aug 25;302(10):113489. doi: 10.1016/j.jbc.2026.113489 (PMC13629211; doi:10.1016/j.jbc.2026.113489)
Supplement: Supplementary file 2 [file mmc2.doc]

**Table S1 OARSI Score**

| **OARSI Score** | | |
| --- | --- | --- |
| **Cartilage**  **Structure** | 0 | Normal |
|  | 1 | Surface Irregularities |
|  | 2 | Pannus and Surface Irregularities |
|  | 3 | Clefts to Transitional Zone |
|  | 4 | Clefts to Radial Zone |
|  | 5 | Clefts to Calcified Zone |
|  | 6 | Complete Destruction |
| **Cell** | 0  1  2  3 | Normal  Hypercellularity (Cell Clusters)  Hypocellularity (Cell Dispersion)  Severe Hypocellularity |
| **Safranin O Staining** | 0  1  2  3  4 | Normal  Slight Loss of Staining  Moderate Loss of Staining  Severe Loss of Staining  No Dye Noted |
| **Tidemark** | 0  1 | Intact  Vascular Invasion (Crossing of the Tidemark) |

**Table S2 Primer Sequences**

| **Name** | **Forward (5'—3')** | **Reverse (5'—3')** |
| --- | --- | --- |
| ***Il-1β*** | TGGACCTTCCAGGATGAGGACA | GTTCATCTCGGAGCCTGTAGTG |
| ***Mmp13*** | CACTTTATGCTTCCTGATGACG | TCTGGCGTTTTTGGATGTTTAG |
| ***β-actin*** | GAAGATCAAGATCATTGCTCCT | TGGAAGGTGGACAGTGAG |
| ***Klf1***  **Promoter** | TGCACAGTGACACTTGGCTT | GGGCTACATAGTCTTTGTCTC |
